# Simultaneous Removal of Lindane, Lead and Cadmium from Soils by Rhamnolipids Combined with Citric Acid

**DOI:** 10.1371/journal.pone.0129978

**Published:** 2015-06-18

**Authors:** Jinzhong Wan, Die Meng, Tao Long, Rongrong Ying, Mao Ye, Shengtian Zhang, Qun Li, Yan Zhou, Yusuo Lin

**Affiliations:** 1 Nanjing Institute of Environmental Science, Ministry of Environmental Protection of China, Nanjing, China; 2 State Environmental Protection Key Laboratory of Soil Environmental Management and Pollution Control, Nanjing, China; 3 State Key Laboratory of Soil and Sustainable Agriculture, Institute of Soil Science, Chinese Academy of Sciences, Nanjing, China; Old Dominion University, UNITED STATES

## Abstract

This study investigated the performance of rhamnolipids-citric acid mixed agents in simultaneous desorption of lindane and heavy metals from soils. The capacity of the mixed agents to solubilize lindane, lead and cadmium in aqueous solution was also explored. The results showed that the presence of citric acid greatly enhanced the solubilization of lindane and cadmium by rhamnolipids. A combined effect of the mixed agents on lindane and heavy metals removal from soils was observed. The maximum desorption ratios for lindane, cadmium and lead were 85.4%, 76.4% and 28.1%, respectively, for the mixed agents containing 1% rhamnolipidsand 0.1 mol/L citric acid. The results also suggest that the removal efficiencies of lead and cadmium were strongly related to their speciations in soils, and metals in the exchangeable and carbonate forms were easier to be removed. Our study suggests that the combining use of rhamnolipids and citric acid is a promising alternative to simultaneously remove organochlorine pesticides and heavy metals from soils.

## Introduction

Organic pollutants and heavy metals are often found together in contaminated soils. At present, 52% of National Priority List sites are co-contaminated by volatile organic compounds, semi-volatile organic compounds and heavy metals in the United States[1]. These contaminated soils are not only a threat to ecological security, but also challenges for remediation projects. Existing methods, such as soil vapor extraction, thermal desorption, phytoremediation and solidification/stabilization are generally only suitable for the removal of either organic pollutants or heavy metals. Thus, remediation technologies for the co-contaminants in soils are urgently required. Soil washing is considered to be a promising alternative for treating organics-heavy metals co-contaminated soils [2,3]. Actually, the simultaneous removal of organics such as polynuclear aromatic hydrocarbons (PAHs) and heavy metals by soil washing has recently attracted increasing attention[4,5].

However, current studies mainly focus on the combination of two chemosynthetic agents that are suitable for either organic pollutants or heavy metals, such as surfactant ssodium dodecyl sulfate (SDS) and ethylene diamine tetraacetic acid (EDTA) [6]. Due to the environmental risk posed by chemosynthetic agents, environmentally friendly agents, including cyclodextrins and biosurfactants, have become more and more attractive in soil remediation [7–11]. In particular, the feasibility of rhamnolipid biosurfactants to remove various hydrophobic organic compounds (HOCs) or heavy metals in soils has been extensively studied [12,13]. Rhamnolipids are mostly produced by *Pseudomonas aeruginosa*,which are composed of one or two rhamnose molecules as a hydrophilic portion, and up to three molecules of hydroxy fattyacids (C_8_-C_14_) as a hydrophobic portion. Abundant evidences have revealed that rhamnolipids can increase the solubilities of HOCs and enhance their desorption and biodegradation rates [14,15]. They also act as ligands and have a strong affinity for metals, such as cadmium and lead, due to their carboxyl groups [16,17]. Thus, rhamnolipids can simultaneously remove HOCs and heavy metals from soils, altough the ability of biosurfactants has not been fully evaluated up to now.

Organic acids, particularly short-chain organic acids (SCOAs), exist ubiquitously in the environment[18]. They can be produced from various biological and chemical processes. Organic acids can form soluble complexes with metal cations and faciliate the mobilization of metals in soils. In fact, the capability of organic acids to extract the heavy metals in soils has been well demonstrated [19,20]. Moreover, researchers recently noticed that the addition of organic acids, such as citric acid or oxalic acid, could enhance the desorption of HOCs from soils [18,21,22]. The result implies that organic acids have the potential to remove mixed contaminants from soils. More interestingly, further investigation reported that the addition of oganic acid could promote the phenanthrene desorption by rhamnolipids[23].

Based on the aforementioned results, a novel and environmentally friendly washing system that combines rhamnolipid biosurfactants with natural organic acids was proposed. It is suggested that this combination has advantages in that 1) the agents are absolutely environmental compatible, and moreover, are favarable to the soil ecological restoration after remediation, which are superior to most thermal or chemical treatments (including soil washing with synthesized chemicals); 2) combined effects on HOCs or heavy metals removal could be anticipated by the mixed system, thereby reducing the remediation cost when compared to conventional washing by individual reagent. However, so far no report could be found on the simultaneous removal of organics and metals in soils by biosurfactant-natural organic acid mixed agents.

The purpose of this study was to investigate the performance of the rhamnolipids-citric acid combined agents for simultaneously removing organochlorine pesticides (OCPs) and heavy metals from simulated soils. Lindane was chosen as the representative OCP, and lead and cadmium were selected as the target heavy metals. The solubilization and desorption of lindane, lead and cadmium by rhamnolipids in the absence or in the presence of citric acid were examined. The speciations of heavy metals in the soils before and after washing was also analyzed. Results of this investigation are expected to provide a theoretical basis and technical support for the simultaneous remediation of HOCs and heavy metals from soils.

## Materials and Methods

### Soil preparation

Clean garden soils were collected from the Nanjing Institute of Environmental Science, China (N 118.83°, S 32.08°). The soils were air-dried and passed through a 2 mm sieve and then blended with lead nitrate and cadmium nitratemixed solutions, with an objective concentration of 500 mg/kg dry soil for lead and cadmium each. After being air-dried and sieved again, an appropriate volume of lindane-hexane solution was added to the soils by continuous mixing. When the solvent had evaporated, the soils were sieved and then stored in a sealed vessel for 6 months. The concentrations of the contaminants were determined before use. The main physiochemical characteristics of the tested soils are given in Table 1.

**Table 1:** Physiochemical properties of the tested soil.

Main properties	Contents
**Particle size (mm)** ^**a**^	
0.2–2.0 (%)	32.7
0.02–0.2 (%)	32.4
0.002–0.02 (%)	28.6
<0.002 (%)	6.3
**pH** ^**b**^	5.1
**Organic matter (g/kg)**	44.3
**CEC (cmol/kg)**	32.8
**Cadmium (mg/kg)**	554.3
**Lead (mg/kg)**	581.1
**Lindane(mg/kg)**	120.1
**USCS classification**	Sandy loam

^a^Hydrometer method by a TM-85 soil densitometer

^b^Soil/water of 1:2.5, with a pH meter (Sartorius PB-21)

### Chemicals

Rhamnolipids (95%) were purchased from Huzhou Gemking Biotechnology Co. Ltd, China. SP Sephadex C-25 and piperazine-N,N′-bis were obtained from Shanghai Kayon Biological Technology Co. Ltd and lindane (>98.5%) was obtained from Dr. Ehrenstorfer GrmbH. Cadmium nitrate, lead nitrate and sodium chloride were all analytically pure and nitric acid and perchloric acid were both in excellent purity.

### Characterization of rhamnolipids

Monomer compositions of rhamnolipids werecharacterized by LC—ESI—MS (Agilent 1100 LC/MSDtrap) [24]. The critical micelle concentration (CMC) of the rhamnolipids was measured by a surface tension meter (JK99B, Zhongchen, Shanghai) using the Du Nouy ring method [25].

### Solubilization of lindane by rhamnolipids and citric acid

Batch solubilization experiments were carried out in triplicate in 40 mL Corex centrifuge tubes with Teflon-lined screw caps. An appropriate volume of lindane-hexane stock solution was added to each tube. When the solvent had evaporated, a series concentration of rhamnolipids and citric acid solutions (pH 7.0±0.1) were added to the tubes. The samples were then agitated in a rotary shaker for 48 h (25±1°C, 200 rpm). After centrifugation at 4000 rpm for 10 min, the supernatant was filtered through a 0.22μm cellulose acetate membrane and then extracted by hexane. The extractant was analyzed for lindane on an Agilent 6890N gas chromatograph equipped with an electron capture detector and a HP-5 capillary column (30.0 mm × 0.32 mm × 0.25 μm). The injector and detector temperatures were 225 and 300°C, respectively, and the injection volume was 1.0 μL. The carrier gas flow rate (99.999% nitrogen) was 1.0 mL/min. The heating procedure was as follows: the oven was heated to 100°C, held for 2 min and thenheated from 100to 195°C at a rate of 20°C/min. Finally, the oven was heated to 270°C at a rate of 3°C/min and then held for 2 min.

### Complexation of heavy metals with rhamnolipids and citric acid

An ion-exchange technique was used to determine the ability of rhamnolipids and citric acid to complex heavy metals[26]. Batch equilibrium experiments were carried out in triplicate in 200 mL HDPE bottles. For each treatment, 10 mL of solutions containing 100 or 1000 mg/L rhamnolipids, 0–0.1 mol/L citric acid, 40 mg/L lead or cadmium ions, 0.1 mol/L PIPES buffer and 100 mg of SP Sephadex C-25 resin,were prepared. The pH of the mixture was adjusted to 7.0 using appropriate concentrations of nitric acidand sodium hydroxide. The samples were equilibrated on a shaker for 2 h (200 rpm, 25±1°C) and allowed to settle for 1 h. After centrifugation and filtration, 4 mL of supernatant and 0.2 mL of concentrated nitric acid were transferred to another bottle and shaken at 100 rpm for 1 h. The concentration of heavy metals in the acidified solutions was determined using an atomic absorption spectrophotometer (PerkinElmer AA 800).

### Enhanced desorption of lindane and heavy metals from soils

Batch desorption experiments were carried out in 40 mL Corex centrifuge tubes. A total of 2.0 g of tested soils were mixed with 20 mL of rhamnolipids and citric acid mixed solutions. The pH of the slurry was adjusted to 7.0±0.1 using appropriate concentrations of nitric acidand sodium hydroxide. The pH value was selected because our prevoius study suggest that at pH 7.0 the rhamnolpids were most efficent to remove lindane, cadmium and lead from soils. The samples were shaken at 200 rpm and 25±1°C for 24 h. After centrifugation and filtration, lindane and heavy metal concentrations in the supernatant were analyzed respectively as described above.

### Speciation analysis of heavy metals in the soils

The tested soils were washed as described above using rhamnolipids and citric acid solutions individually or in combination. Then the metal distribution in the treated soils was measured using the sequential extraction procedure developed by Tessier et al.[27]. The metal species in the soils were fractionated as exchangeable fractions, carbonate bound fractions, iron and manganese oxides fractions, organically-bound fractions and residual fractions. The concentration of each fraction was determined on the atomic absorption spectrophotometer.

## Results and Discussion

### Characterization of rhamnolipids

As identified by the LC-ESI-MS, the rhamnolipids used herein were primarily consisted of four composites, wherein the fraction of the monorhamnolipids was 83.6%. In particular, RhC_10_C_10_ accounted for the largest fraction of 67.2%, followed by Rh_2_C_10_C_10_(16.4%), RhC_10_C_8_ (7.4%) and RhC_12_C_10_ (7.0%). The average relative molecular weight of the rhamnolipids mix was estimated as 523.7 according to the information of monomer composition. Furthermore, the CMC value of the rhamnolipid mix was determined as 50.0 mg/L, about 0.01 mmol/L.

### Solubilization of lindane in the rhamnolipids-citric acid combined system

**Fig 1:**
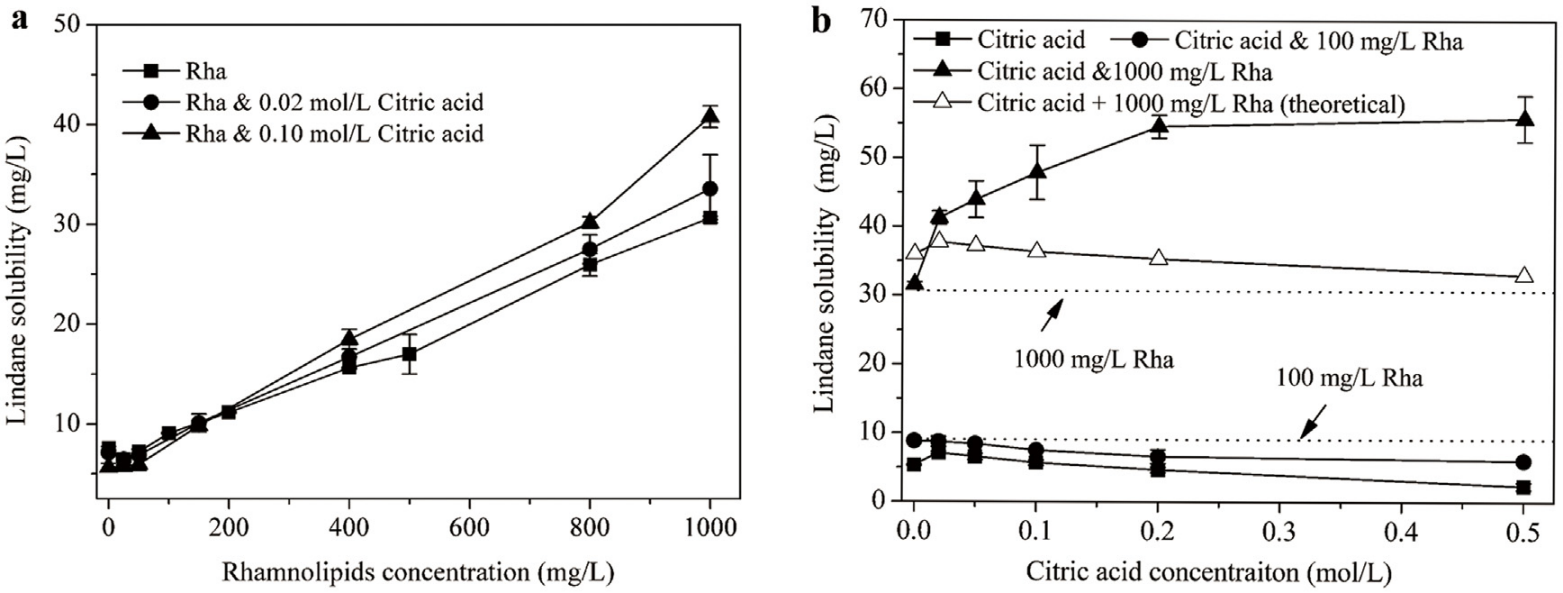
Lindane solubilization as a function of (a) rhamnolipids and (b) citric acid concentration in the rhamnolipids-citric acid combined system. Rha denotes rhamnolipids.

The dissolution behaviors of lindane in rhamnolipids and citric acid alone as well as mixed solutions were investigated. Inspection of Fig 1a reveals that the solubility of lindane increased linearly as a function of rhamnolipids concentration for either individual or mixed agents. Furthermore, the differences between the three curves indicate a considerable enhancing effect of citric acid on lindane solubilization.

Molar solubilization ratio (MSR) is generally used to evaluate the solubilization effect of surfactants, which is defined as[28]:

$$\text{MSR}=\frac{C_{\text{HOC, mic}}-C_{\text{HOC, cmc}}}{C_{\text{surf}}-CMC}$$

Where C_surf_ is the concentration of surfactant added (mol/L), and C_HOC,mic_, C_HOC,cmc_ are the apparent solubilities of HOCs (mol/L) in surfactant solutions at concentrations above CMC and at CMC, respectively. By using above equation, the MSR value with rhamnolipids alone was 0.043. Meanwhile, the MSR values for rhamnolipids plus 0.02 and 0.1 mol/L citric acid were 0.048 and 0.061, respectively, which were increased by 11.6% and 41.8% respectively relative to rhamnolipids alone.

The stimulative effect of co-present chemicals on HOCs solubilization was also mentioned in previous studies. Cao et al. [5] reported that the lingand S, S-ethylenediaminedisuccinic acid (EDDS) could enhance the solubilization of PCBs by saponin. In another study by Zhou et al.[28], the addition of cadmium and zinic ions promoted the phenanthrene solubilization by saponin. The results were attributed to the reduced CMC values as an effect of EDDS or metal ions addition[5, 28]. In this study, increasing ionic strength was expected especially at high concentrations of citric acidor rhamnolipids when the system pH was adjusted to 7.0. As a consequence, the CMC values of rhamnolipids would be lowered, and more lindane was dissolved[5, 28]. In addition, it has been documented that polar organics could enter the palisades layer of the micelles and enlarge the size of micelles, which provides larger hydrophobic interspace for HOCs molecules [29]. The promotive effect of the combined system herein might be also associated with above interaction between citric acid and rhamnolipids surfactants.

Fig 1b illustrates the solubilization of lindane by mixed agents as a function of citric acid concentration. Unexpectedly, for citric acid alone system, lindane solubility declined slightly with increasing citric acid concentration. Correspondingly, the mixed solution (with 100 mg/L rhamnolipids) exhibited increasingly lower solubilization effect compared to rhamnolipids alone. In contrast, at a high rhamnolipds concentration of 1000 mg/L, lindane solubility increased substantially when citric acid concentration ranged from 0 to 0.2 mol/L, then reached a plateau although citric acid further increased to 0.5 mol/L. The higher solubilization curve for mixed agents containing 1000 mg/L rhamnolipids than rhamnolipids alone confirms the promotive effect of citric acid on lindane dissolution. Furthermore, note that the observed solubilization curve for mixed agents (Citric acid & 1000 mg/L Rha) was significantly higher than the theoretical curve (Citric acid + 1000 mg/L Rha, plotting by the sum of lindane solubility in citric acid and rhamnolipids individual solution), which implies a reliable synergistic effect on lindane solubilization by the mixed system. Particularly, Table 2 indicates that the absolute and relative synergistic increments of lindane solubilization brought by the combined agents were 3.52–22.8 mg/L and 9.32–69.1%, respectively. Moreover, the synergistic effect was enhanced with the citric acid concentration increasing. Similarly, we previously reported a synergistic percentage of 29–132% on hexachlorobenzene solubilization when cyclodextrin was combined with 30% ethanol[30].

**Table 2:** Synergistic effect of the mixed agents on lindane solubilization.^a^

Synergy	Citric acid concentration, mol/L					
	0	0.02	0.05	0.10	0.20	0.50
**Absolute (mg/L)**	0	3.52	6.73	11.5	19.2	22.8
**Relative (%)**	0	9.32	18.1	31.8	54.5	69.1

^a^ At fixed rhamnolipids concentration of 1000 mg/L.

### Complexation of heavy metals with rhamnolipids and citric acid

**Fig 2:**
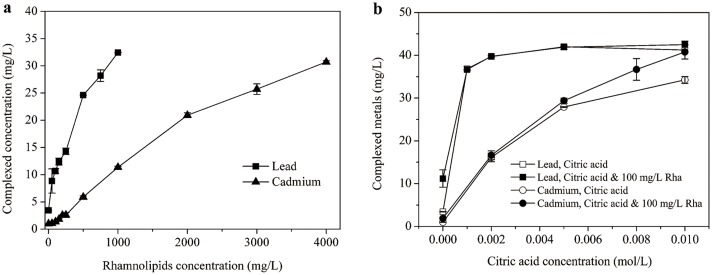
Lead and cadmium complexation in (a) rhamnolipidsand (b) Rhamnolipids-citric acid mixed solutions.

The complexation behaviors of lead and cadmium in rhamnolipids and citric acid individual and mixed systems were investigated and the results are shown in Fig 2. The aqueous lead and cadmium concentrations were 0.43 and 0.98 mg/L, respectively, when neither rhamnolipids nor citric acid was added,which means approximately 91.4% of lead and 97.5% of cadmium were bound to the SP Sephadex C-25 resin. It can be found from Fig 2a that the aqueous lead and cadmium concentrations increased nearly linearly as rhamnolipids concentration increased. As documented, rhamnolipids can bond with heavy metals and form dissolved complexes by their carboxyl groups of micelle[10]. Furthermore, the complexation mole ratios for Pb/RL and Cd/RL were calculated as 0.2 and 0.05, respectively, revealing that lead complexed more readily with rhamnolipids than cadmium. The results agree well with those by Herman et al. [31], wherein the complexation of three metals to rhamnolipids was in the order of Pb^2+^> Cd^2+^≈Zn^2+^. Ochoa-Loza et al. [26]further reported that the order of rhamnolipid stability constants for the metals was Al^3+^>>Cu^2+^>Pb^2+^>Cd^2+^>Zn^2+^>Fe^3+^>Hg^2+^>Ca^2+^>Co^2+^>Ni^2+^>Mn^2+^>Mg^2+^>K^+^.

Inspection of Fig 2b reveals that citric acid had a rather strong affinity to lead. When the citric acid concentration was 0.001 mol/L, over 92% of lead was complexed. As a result, insignificant difference in aqueous lead concentrationwas observed between rhamnolipids-citric acid mixed solution and citric acid alone. In the case of cadmium, the complexed concentration of which was directly depended on citric acid concentration, whereas negligible cadmium complexation occurred for rhamnolipids alone. Furthermore, an appreciable combined effect on cadmium complexation was attained by the co-presence of 100 mg/L rhamnolipids especially at higher citric acid concentrations (> 0.008 mol/L). By measuring the octanol-water partition coefficients of metals in EDDS/saponin system, Cao et al. [5]also observed an enhanced complexation of lead and copper with saponin by the addition of EDDS. Moreover, it was revealed that lead bonded more strongly than cadmium to rhamnolipids and citric acid in either individual or mixed system. According to the literature, the stability constants for citric acid-lead and citric acid-cadmium are 5.40 and 4.10[32], and those for rhamnolipids-leadand rhamnolipids-cadmium are 8.58 and 6.89, respectively [31]. As demonstrated, lead is a soft Lewis acid, which will form more stable complexes with ligands than some other metal ions, such as cadmium and zinc[26].

### Simultaneous removal of lindane and heavy metals from soils by mixed agents

#### Desorption of lindane

**Fig 3:**
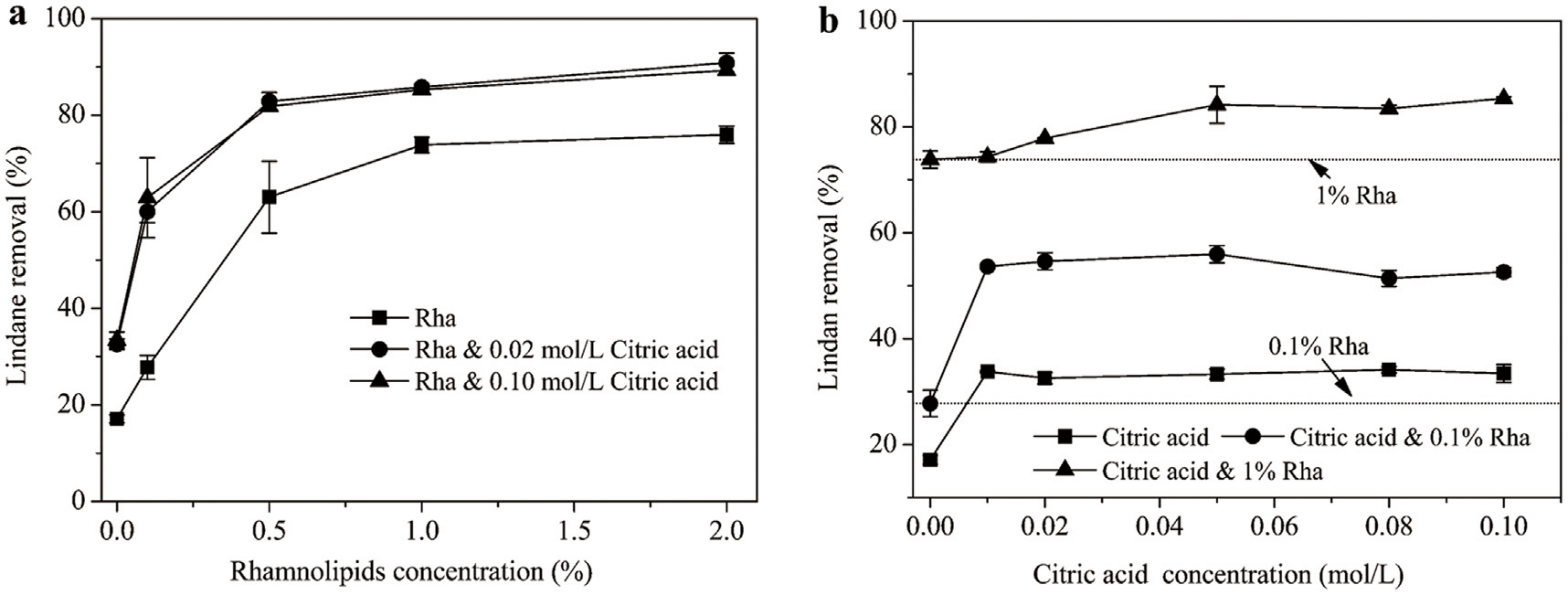
Lindane removal from co-contaminated soils by rhamnolipids-citric acid mixed agents as an effect of (a) rhamnolipids and (b) citric acid concentration.

Fig 3 displays the removal efficacies of lindane from soils by rhamnolipids and rhamnolipids-citric acid mixed solutions. The application of rhamnolipids greatly promoted the desorption of lindane. Particularly, the removal ratio of lindane increased substantially from 17.1% to 76.0% in the rhamnolipids concentration range of 0–10000 mg/L, and kept relatively stable with rhamnolipids further increasing to 20000 mg/L (Fig 3a). Similar trends were also observed for mixed agents with 0.02 and 0.10 mol/Lcitric acid. Furthermore, significant higher desorption ratios of lindane were achieved by mixed agents compared to rhamnolipids alone, indicating a reliable combined effect by the presence of citric acid. The increments of lindane removal ratio was in the range of 12.0–32.3% and 11.5–35.1%, respectively, when 0.02 and 0.10 mol/L citric acid were combined with varied dosages of rhamnolipids. The promotive effect of short-chain organic acids on rhamonolipids-enhanced mobilization of phenanthrene has been evidenced by An et al.[23], wherein the combination of rhamnolipids and citric acid increased the desorption amounts of phenanthrene by 1.09–2.24 times. Cao et al. [5] also reported a synergy on PCB-5 desorption by coupling use of chelating agent EDDS and biosurfactants saponin.

The mechanism that might contribute to the combined effect of rhamnolipids- citric acid mixed agents on the lindane desorption is rather complex. As well known, the adsorption of HOCs to soils is mainly controlled by their partitioning to soil organic matter (SOM)[33]. An et al. [23] proposed that the low molecular organic acids (including citric acid) could break the SOM-metal-mineral linkages by forming organically complexed metals, thus promoting the release of SOM. Consequently, the dissolution of contaminants was enhanced along with the desorbed SOM [23]. However, the effect of HOCs solubilization by mixed agentson the desorption of HOCs has not been mentioned in prevoius studies. In fact, micelle solubilization is a crucial process for the enhanced desorption of HOCs by surfactants from soils[28]. In this sense, the combined effect of mixed agents on the lindane desorption could be also explained by the reliable synergy on lindane solubilization (see Section 3.3). Moreover, it is suggested that citric acid could occupy the adsorptive sites of the soil particle surface and reduce the adsorption of both rhamnolipids and lindane to the soil matrix[5,23].

Fig 3b shows the removal of lindane by mixed agents as a function of citric acid concentration. The trends for citric acid alone and citric acid with 0.1% rhamnolipids were very similar. A steep increase in lindane desorption was observed with citric acid concentration rising from 0 to 0.01 mol/L, which indicates an appreciable stimulation of contaminant desorption by citric acid. However, further increasing the citric acid concentration above 0.01 mol/L resulted in no significant improvement in lindane removal. The enhanced mobilization of PAHs in soils by organic acids has been well demonstrated[18,23,34]. As noted, the organic acids can bind soil metal cations and enhance the release of SOM, resulting in more absorbed contaminated dissolved[23]. For the combination of citric acid and 1% rhamnolpids, the lindane desorption ratios increased gradually over the whole citric acid concentration range. An et al. [23] also observed that the removal of phenanthrene by rhamnolipids mixed with organic acids increased gradually as the organic acids concentration increased to 300 mmol/L. The higher the citric acids added, the more organics acids available to destroy the barriers within soil matrix that might impede the mobilization of HOCs[23]. Furthermore, the remarkable difference between the desorption curves for citric acid and citric acid & 1% rhamnolipids (Fig 3b) illustrates a predominant role of rhamnolipids in lindane desorption from soils. The conclusion can be confirmed by Fig 3a, wherein 1% rhamnolipids acounted for 51.3–86.5% of the lindane desorption.

#### Desorption of lead and cadmium

**Fig 4:**
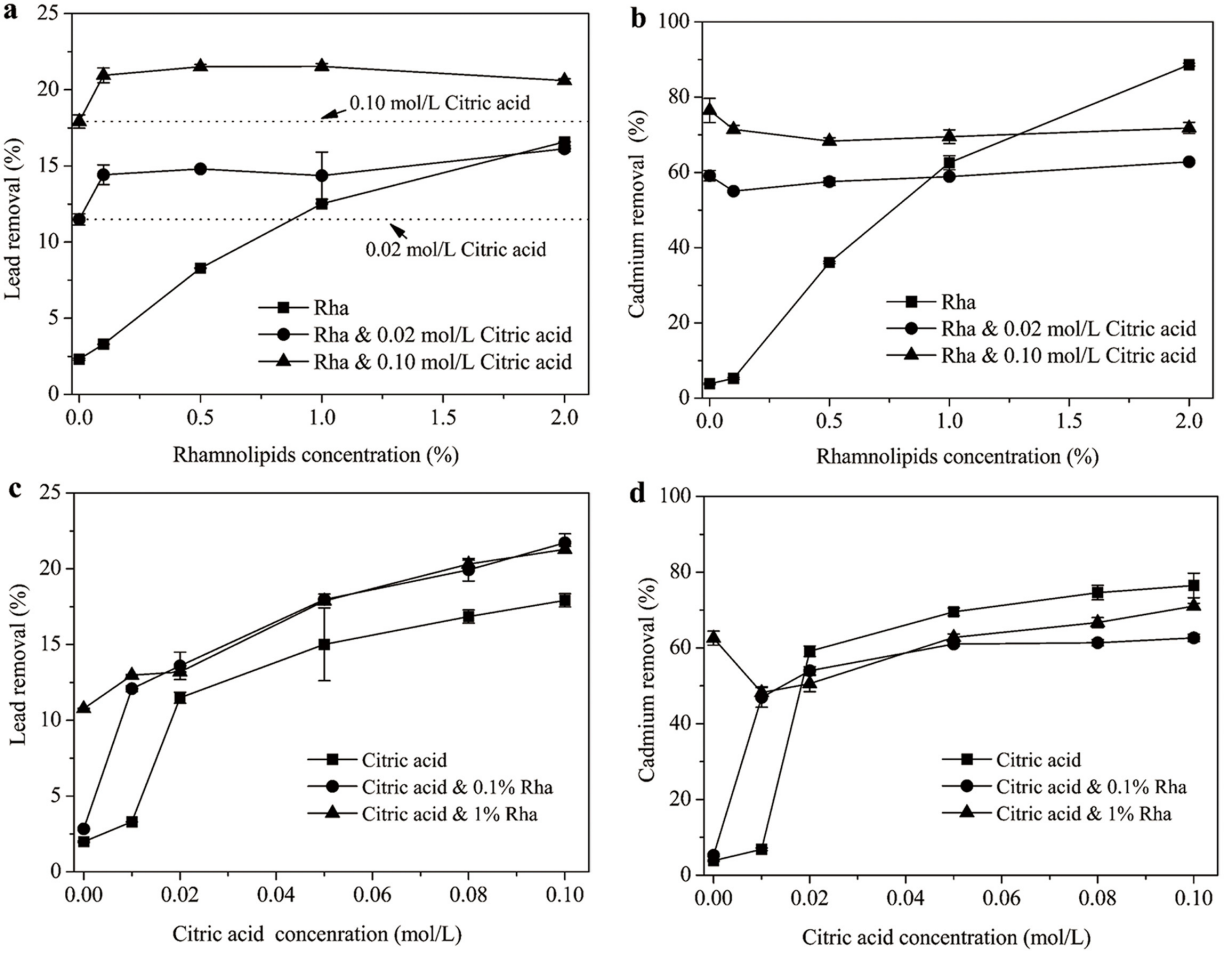
Lead and cadmium removal from co-contaminated soils by rhamnolipids-citric acid mixed agents. (a) Lead and (b) cadmium removal as an effect of rhamnolipids concentration. (c) Lead and (d) Cadmium removal as an effect of citric acid concentration.

Fig 4 illustrates the desorption behaviors of lead and cadmium in soil-water system when rhamnolipids and citric acid were applied individually or in combination. In the absence of citric acid, the lead removal increased with increasing rhamnolipids concentration. Comparatively, when citric acid was present, the lead desorption was greatly promoted regardless of the rhamnolipids concentration. For instance, for mixed agents containing 0.02 and 0.10 mol/L citric acid, the desorption ratios of lead were 14.8 and 21.5%, respectively, relative to 8.3% for 0.5% rhamnolipids alone. Furthermore, note that the removal ratio of lead by mixed agents was higher than that by citric acid alone (Fig 4a), which reveals an appreciable combined effect associated with the mixed agents. The result also implies that both rhamnolipids and citric acid contributed to the enhanced desorption of lead. In a study by Slizovskiy et al.[35], obviously enhanced desorption of heavy metals (Pb, Cu, Zn, Cd) was observed when citrate buffer (pH 3.6) or EDTA was added to chemical or bio-surfactants (rhamnolipids JBR-425) solutions. Ehsan et al. [36] also reported that lead and copper mobilization was greatly increased by the combination of EDTA and surfactants (nonionic Brij 98 and Triton XQS-20). Generally, the decreased adsorption of citric acid due to the presence of rhamnolipids, and the enhanced dissolution of SOM by citric acid and rhamnolipids and the subsequent forming DOM-lead complex were probably responsible for the combined effect of the mixed agents [23].

Fig 4b shows that cadmium removal ratio was also positively related to biosurfactant concentration for rhamnolipids alone system. Over 90% of cadmium could be desorbed from soils by 2% rhamnolipids. Different from lead, the presence of citric acid exhibited a dual effect on cadmium desorption when coupling it to rhamnolipids. At lower rhamnolipids concentrations (below 1.0%), organic acid stimulated cadmium desorption, whereas further increasing rhamnolipids concentrations to 2% resulted in an inhibitation for cadmium removal.

The effect of citric acid concentration on the desorption of lead and cadmium by mixed system was also studied. As shown in Fig 4c, for either mixed or individual agent, the desorption ratio of lead was directly correlated with the concentration of citric acid. Moreover, mixed agents (containing 0.1% or 1% rhamnolipids) had higher lead desorption performances than citric acid alone, confirming the combined effect of the mixed agents on lead desorption (Fig 4c). However, inspection of Fig 4d indicates that compared to citric acid alone, the cadmium desorption was slightly hindered for the mixed agents especially for citric acid with 0.1% rhamnolipids. The reason may be that for the mixed agents system, more lead was desorbed from soils than individual agent (Fig 4b), which reduced the competitive adsorption between lead and cadmium to soils [31].

In general, for either individual or mixed agents, the desorption ratio of lead was much lower than that of cadmium. The mobilization of cadmium by either acid or chelating agents is easier than that of lead and chromium, which has been evidenced by a number of studies[31,36]. As stated, lead is a soft Lewis acid, and cadmiumisthe borderline between soft and hard, which meanslead will form more stable complex with SOM and adsorb morestrongly to soils than cadmium ions[31].

### Speciations of heavy metals in soils

Sequential extraction experiments were performed on the soils before and after washing to determine which fractions were removed. Although the distribution of heavy metals as analyzed by sequential extraction does not necessarily reflect their association with discrete soil phases, it has been widely used to measure the bioavailability and environmental risk of heavy metals as well as to evaluate the remediation performance[37, 38]. The fractionation of the tested soils in Table 3 indicates that Fe-Mn oxides and the residual forms were the predominant species for lead, the percentages of which were 36.2% and 29.1%, respectively. The exchangeable form, whichis documented as the most mobilizable in soils, only accounts for 15.1%. To the contrary, soil cadmium existed mostly in the exchangeable form, which accounts for 89.2% of the total amount in the tested soils.

**Table 3:** Fractionation of metals in tested soils.

Metal	Fraction (% of total)				
	Exchangeable	Carbonates	Fe-Mn Oxides	Organic	Residual
**Lead**	13.0	6.1	36.3	15.4	29.1
**Cadmium**	89.2	3.9	5.2	1.3	0.4

**Fig 5:**
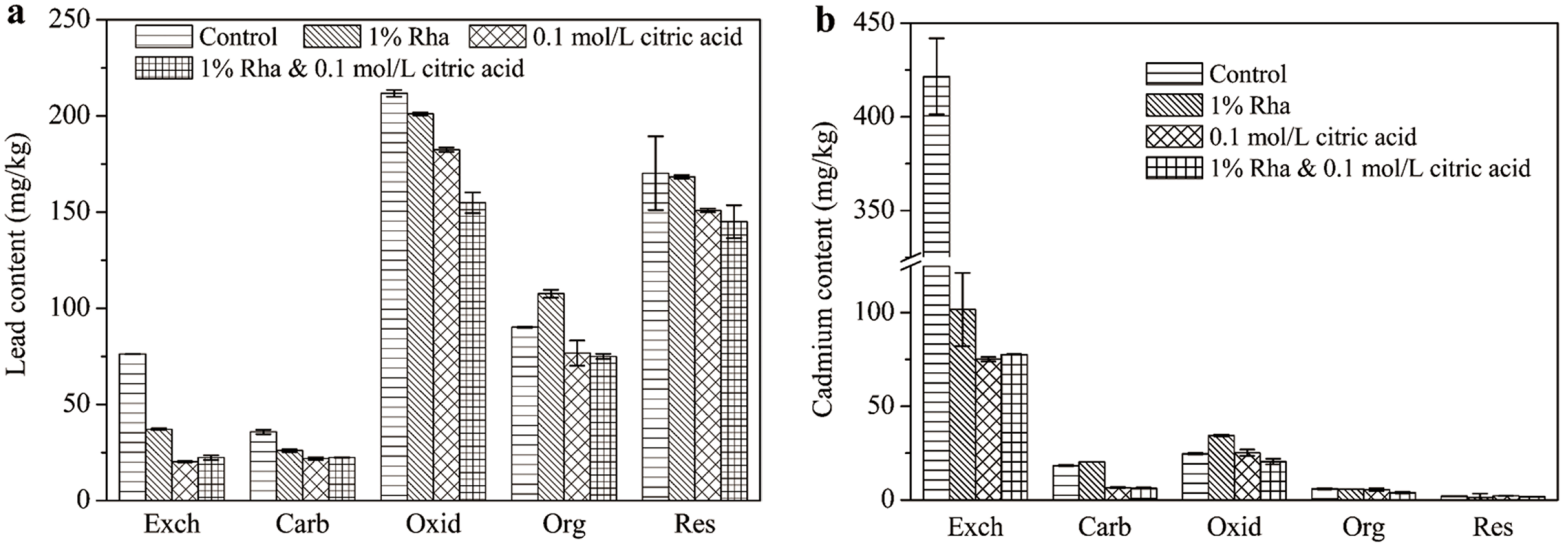
Fractionation of (a) lead and (b) cadmium in soils before and after washing treatment. Exch, Carb, Oxid, Org, Res denote the heavy metals in the exchangeable, carbonates, Fe-Mn oxides, organic and residual forms, respectively.

Fig 5 depicts the fractionation of heavy metals in soils before and after the different washing treatments. It was suggested that the exchangeable, carbonate, and Fe-Mn oxides forms can beeasily dislodged by the washing procedures[36]. For lead, the exchangeable form was the most readily removed (reduced by 51.3–73.4%), followed by the carbonate form (27.3–38.9%). The Fe-Mn oxides, organically bound and residual fractions, which existed as the dominant proportions of the lead, were generally reduced by lower than 20%. As a result, seemingly inefficient removal was found for lead even though the mixed agents were used (Fig 4a and Table 4). It can be inferred from Table 4 that citric acid was more effective to detach most of the lead forms than rhamnolipds. Furthermore, the combination of rhamnolipids and citric acid could further enhance the removal of the relatively immobilized fractions especially the Fe-Mn oxides, which may be responsible for the combined effects of mixed agents on lead extraction (Fig 4a). As for cadmium, the exchangeable fraction was also easiest to be removed, with desorption ratios of 75.9–82.2% when rhamnolipids and citric acid were used individually or in combination, which could explain the reliable desorption of soil cadmium. The cadmium in the carbonate form was also significantly reduced by citric acid and mixed agents, coincident with lead. Table 4 also suggests that citric acid played a dominant role in the enhanced desorption of lead and cadmium in the combined agents system, which is consistent with the findings of Cao et al. [5], wherein the lingand EDDS functioned primarily for lead and copper desorption relative to saponin biosurfactant. Moreover, Mulligan et al. [37] stated that any form, other than the residualform, could be removed by chemically enhanced soil washing. The results in Table 4 are supposed to support this finding.

**Table 4:** Removal percentages of different metal species after washing treatments.^a^

Fractionation	Lead			Cadmium		
	Rha^b^	CA^c^	Rha & CA^d^	Rha	CA	Rha & CA
**Exchangeable**	51.3	73.4	70.7	75.9	82.2	81.6
**Carbonates**	27.3	38.9	37.4	-10.3	64.1	65.3
**Fe-Mn Oxides**	5.0	13.9	26.8	-39.5	-2.2	13.3
**Organic**	-19.3	15.0	16.8	2.5	6.7	33.1
**Residual**	1.1	11.3	14.8	-37.8	-7.6	10.1
**Total**	7.5	22.6	28.1	65.1	75.7	76.4

^a^Calculated by dividing the difference between the final (afte washing) and initial (before washing) species conents in soils by the initial value.

^b^ Rhamnolipids, 1%;

^c^ Citric acid, 0.1 mol/L;

^d^ 1% Rhamnolipids plus 0.1 mol/Lcitric acid.

## Conclusions

The rhamnolipids-citric acid mixed agents exhibited substantial combined or synergistic effect on cadmium and lindane solubilization, and superior extraction efficacy for co-contaminants from soils when compared to using them separately. Sequential extraction experiments revealed that both the combined effect of mixed agents on lead removal and the differentiated removal ratios between lead and cadmium were correlated with the fractionation of heavy metals. The exchangeable and carbonates fractions were more readily to be removed. Overall, our results proved that the combined use of rhamnolipids and citric acid could simultaneously remove OCPs and heavy metals from soils. Moreover, as aforementioned, unlike most of the physical or chemical methods, the remediation process is totally environmentally friendly, with little or no negaitve impact to the environment. Moreover, it should also be noted that compared to synthesized funtional materials for mixed contaminants (such as N,N-bis-dodecyl-S,S-EDDS [2]), both of the two reagents herein (rhamnolipids and citric acid) are commercial avialable, which offers higher applicability of the rhamnolpipids-citric acid combination in the environmental remediation. Overall, it is reasonable to suggest that enhanced washing with the rhamnolpipids-citric acid mixed agents would be a promising alternative for the remediation of soils with HOCs and heavy metals.
